# Efficient high-throughput molecular method to detect *Ehrlichia ruminantium* in ticks

**DOI:** 10.1186/s13071-017-2490-0

**Published:** 2017-11-13

**Authors:** Nídia Cangi, Valérie Pinarello, Laure Bournez, Thierry Lefrançois, Emmanuel Albina, Luís Neves, Nathalie Vachiéry

**Affiliations:** 1 CIRAD, UMR ASTRE, F-97170 Petit-Bourg, Guadeloupe France; 2 ASTRE, Univ Montpellier, CIRAD, INRA, Montpellier, France; 3 Centro de Biotecnologia-UEM, Universidade Eduardo Mondlane, Maputo, Mozambique; 4 Université des Antilles, Pointe à Pitre, Guadeloupe France; 5 CIRAD, UMR ASTRE, F-34398 Montpellier, France; 6 Department of Veterinary Tropical Diseases, University of Pretoria, Faculty of Veterinary Science, Onderstepoort, South Africa

**Keywords:** *Ehrlichia ruminantium*, Ticks, *pCS20*, Automated DNA extraction, qPCR

## Abstract

**Background:**

*Ehrlichia ruminantium* is the causal agent of heartwater, a fatal tropical disease affecting ruminants with important economic impacts. This bacterium is transmitted by *Amblyomma* ticks and is present in sub-Saharan Africa, islands in the Indian Ocean and the Caribbean, where it represents a threat to the American mainland.

**Methods:**

An automated DNA extraction method was adapted for *Amblyomma* ticks and a new qPCR targeting the *pCS20* region was developed to improve *E. ruminantium* screening capacity and diagnosis. The first step in the preparation of tick samples, before extraction, was not automated but was considerably improved by using a Tissue Lyser. The new *pCS20* Sol1 qPCR and a previously published *pCS20* Cow qPCR were evaluated with the OIE standard *pCS20* nested PCR.

**Results:**

*pCS20* Sol1 qPCR was found to be more specific than the nested PCR, with a 5-fold increase in sensitivity (3 copies/reaction vs 15 copies/reaction), was less prone to contamination and less time-consuming. As *pCS20* Sol1 qPCR did not detect *Rickettsia*, *Anasplasma* and *Babesia* species or closely related species such as Panola Mountain Ehrlichia, *E. chaffeensis* and *E. canis*, its specificity was also better than Cow qPCR. In parallel, a tick 16S qPCR was developed for the quality control of DNA extraction that confirmed the good reproducibility of the automated extraction. The whole method, including the automated DNA extraction and *pCS20* Sol1 qPCR, was shown to be sensitive, specific and highly reproducible with the same limit of detection as the combined manual DNA extraction and nested PCR, i.e. 6 copies/reaction. Finally, 96 samples can be tested in one day compared to the four days required for manual DNA extraction and nested PCR.

**Conclusions:**

The adaptation of an automated DNA extraction using a DNA/RNA viral extraction kit for tick samples and the development of a new qPCR increased the accuracy of *E. ruminantium* epidemiological studies, as well as the diagnostic capabilities and turn-over time for surveillance of heartwater. This new method paves the way for large-scale screening of other bacteria and viruses in ticks as well as genetic characterization of ticks and tick-pathogen coevolution studies.

**Electronic supplementary material:**

The online version of this article (10.1186/s13071-017-2490-0) contains supplementary material, which is available to authorized users.

## Background

*Ehrlichia ruminantium* is an obligate intracellular bacterium that causes heartwater, an infectious, virulent, transmissible but non-contagious disease of ruminants [1]. Its main vectors are *Amblyomma hebraeum* ticks in southern Africa and *A. variegatum* ticks that transmit the disease to the rest of sub-Saharan Africa, and to islands in the Indian Ocean and the Caribbean [2, 3]. Heartwater is one of the major obstacles to the introduction of high-producing animals to upgrade and replace local stock in Africa [4]. Its economic impact is high, estimated at US $44.7 million per year for the SADC region (Southern Africa Development Community) [5]. The U.S. Homeland Security Department for the American mainland lists heartwater as one of the 12 most important animal transboundary diseases [6]. Effective vaccines to control the disease are needed [1], but to date, experimental vaccines including recombinant, attenuated and inactivated vaccines have not been particularly successful, presumably owing to the antigenic variability of the pathogen [7, 8]. In this context, characterization of field strains is indispensable to design appropriate vaccines including regional strains.

Ideally, a large number of ticks need to be collected and tested to accurately evaluate the prevalence of *E. ruminantium* in ticks from endemic areas, and further characterize the genetic diversity and population structure of *E. ruminantium* from several geographical areas [9, 10]. In addition, given that certain areas (e.g. the American mainland) are at high risk of the introduction of heartwater, it is advantageous to have rapid high-throughput molecular tools in preparation for the possible introduction of the pathogen in a previously pathogen-free area. Various methods for manual extraction of tick DNA are currently available and result in high DNA yields [11, 12]. However, all these methods have a low sample processing capacity and are time consuming. A few automated DNA extraction methods have been tested for arthropods including spiders and flies [13–15]. Moriarity et al. [16] developed a high-throughput DNA extraction method specifically for ticks, including *Ixodes scapularis* and optimized a qPCR for the detection of *Rickettsia rickettsii*, *R. sibirica*, *R. africae* and *R. prowazekii* using the Promega Wizard SV96 genomic DNA purification system [16]. Crowder et al. [17] automated a Qiagen (Courtaboeuf, France) MiniElute Virus extraction kit and detected the presence of *Borrelia burgdorferi* and Powassan virus in *I. scapularis* ticks [17].

Several molecular methods targeting different specific *E. ruminantium* genes or regions have been developed to diagnose heartwater in ruminants and to screen *E. ruminantium* in ticks. Some of these methods target the *pCS20* region, a highly conserved and specific gene region of *E. ruminantium*. The *pCS20* nested PCR, recommended by OIE, the World Organization for Animal Health, has been tested on a wide range of *E. ruminantium* strains from cattle and tick samples [18–20]. However, it has two main disadvantages: it is time consuming and the risk of contamination is high. To solve these problems and to address the need for quantitative results, qPCRs have been developed for the detection and quantification of *E. ruminantium*. A SYBR Green and TaqMan qPCRs targeting two different regions of the *map-1* gene were used to quantify *E. ruminantium* during vaccine production and growth in endothelial cell culture [21, 22]. Likewise, *E. ruminantium map1–1* transcripts were quantified in tick midguts and salivary glands as well as in *E. ruminantium* infected endothelial cell cultures by SYBR Green RT-qPCR targeting the *map-1*-*1* gene [23]. However, due to the polymorphism of the *map1* multigenic family, assays targeting these genes are not suitable for diagnostic tests. A *pCS20* quantitative real-time PCR based on a TaqMan probe, Cow^TqM^, was developed in 2008 by Steyn et al. [24] to detect *E. ruminantium* in livestock blood and ticks from the fin l [24]. However, Cow^TqM^ qPCR cross-reacts with *E. chaffeensis* and *E. canis*. Since *E. chaffeensis* is widespread in the USA and *E. canis* or related species, have recently been observed in the Caribbean [25], Cow^TqM^ qPCR cannot be used as a diagnostic tool in mainland USA and in the Caribbean. Nakao et al. [26] developed another method for rapid low cost detection of *E. ruminantium* using loop-mediated isothermal amplification (LAMP) targeting the *pCS20* and *sodB* gene regions, and tested it on blood samples and ticks [26]. However, this technique, which was tested on 16 *E. ruminantium* strains, was less sensitive than Cow^TqM^ qPCR due to the inhibitory effects of *A. variegatum* ticks. These results prevented the use of this LAMP method for the detection of *E. ruminantium* in ticks and no additional data have become available since its publication in 2010.

In addition, multi-pathogen qPCRs including *E. ruminantium* detection have been developed. Sayler et al. [27] developed and validated a dual-plex TaqMan qPCR assay targeting the *groEL* gene of Panola Mountain Ehrlichia (PME) and *E. ruminantium* in field samples from ruminants or from ticks, which enabled the differentiation of the two species in the USA [27]. A generic *Ehrlichia* FRET-qPCR targeting the *16S* rRNA gene has also been developed [25]. Based on melting point analysis, this method made it possible to distinguish eight *Ehrlichia* species from four groups: *E. ruminantium* (Group 1); *E. chaffeensis* and *E. ewingii* (Group 2); *E. canis*, *E. muris*, *E. ovina* and *Ehrlichia* sp. BOV2010 (Group 3) and PME (Group 4).

Since all these techniques failed to address the question of the scalability of tick sample processing and screening, we decided to adapt a commercial automated DNA extraction kit to ticks and to develop a new qPCR assay with improved specificity and sensitivity compared to the OIE standard nested PCR and the published Cow^TqM^ qPCR. To this end, a high-throughput DNA extraction method and a new *pCS20* Sol1 qPCR assay were optimized.

The new *pCS20* Sol1 qPCR (both, SYBR Green, Sol1^SG^ qPCR and TaqMan, Sol1^TqM^ qPCR), the previously published Cow^TqM^ qPCR and the OIE standard *pCS20* nested PCR were evaluated. In parallel, a tick 16S rDNA qPCR was developed to check the quality of tick DNA extraction and the absence of PCR inhibitors. The whole method, including automated DNA extraction and Sol1^TqM^ qPCR, was then compared to manual DNA extraction and nested PCR reference methods. The advantages of this new qPCR over those already published are discussed.

## Methods

### Development of *pCS20* Sol1^TqM^ and Sol1^SG^ qPCRs

#### Design of pCS20 Sol1 primers and probes

For the design of Sol1 primers and probes, we identified the most conserved gene region of *E. ruminantium*, *pCS20*, through multiple alignments of nucleotide sequences from 13 strains available in GenBank (detailed in Additional file 1).

We developed the new *pCS20* Sol1 qPCR using two types of chemistry, one based on SYBR Green ^(SG)^ and the second using the TaqMan ^(TqM)^ technology. The cycling conditions are described in detail in Additional file 1: Table S1.

#### Efficiency, limit of detection and reproducibility

*Ehrlichia ruminantium* from the strain Gardel passage 48, was grown in bovine aorta endothelial cells as previously described [28]. DNA was then extracted using the QiaAmp DNA minikit (Qiagen, Courtaboeuf, France) according to the manufacturer’s instructions and following the protocol of Frutos et al. [29]. The extracted DNA was quantified by a *map-1* ^TqM^ qPCR [22]. We tested 10-fold serial dilutions of *E. ruminantium* ranging from 3 × 10^6^ to 30 copies/reaction in triplicate with Sol1^TqM^ and Sol1^SG^ qPCRs at annealing temperatures ranging from 48 °C to 56 °C, in order to optimize the qPCR efficiency as previously described [30].

At optimal temperatures, we determined the limit of detection of both Sol1 qPCRs in testing the same serial dilutions of *E. ruminantium* Gardel DNA passage 48 and an additional sample with 3 copies/reaction. To comparatively assess the performance of the new *pCS20* Sol1 qPCRs, the limit of detection was also determined for the conventional *pCS20* nested PCR and *pCS20* Cow^TqM^ qPCR as described by Steyn et al. [24]. For the nested PCR, only 1 μl of DNA was run instead of 2 μl for the two qPCRs, with final concentrations ranging from 1.5 × 10^6^ to 1.5 copies/reaction. For *pCS20* Cow^TqM^, the running temperature of 48 °C recommended by the authors was used and 56 °C, which is close to the theoretical annealing temperature of its probe, was also tested [24]. The detailed cycling conditions are described in Additional file 1: Table S1.

#### Analytical sensitivity and specificity

We evaluated the analytical sensitivity of the *pCS20* Sol1^TqM^ qPCR with DNA extracted from 16 *E. ruminantium* strains isolated in different geographical areas (Sudan, Burkina Faso, Senegal, South Africa, Zambia, Ghana, Cameroon, Mozambique and Guadeloupe) (Table 1) [31, 32]. In addition, we tested 10 isolates from South Africa (Kruger National Park) and Mozambique [three sites from Maputo Province: Matutuine (MAT), Chobela (CHOB) and Changalane (CHA)], collected in a previous study [33].

**Table 1:** *pCS20* Sol1^TqM^ qPCR sensitivity and specificity test on *Ehrlichia ruminantium* strains and other tick-borne pathogens

Name	Origin	Geographical origin	Nested *pCS20* PCR	Sol1^TqM^ QPCR	Reference
*E. ruminantium* strains					
Blonde	CC p8	Guadeloupe	+	+	[31]
Gardel	CC p48		+	+	[29]
Sara 455	CC p10	Burkina Faso	+	+	[8]
Bekuy 255	CC p9		+	+	[31]
Bankouma 421	CC p15		+	+	[8]
Banankeledaga	CC p1		+	w+	[8]
Lamba 479	CC p16		+	+	[31]
Cameroun	CC p9	Cameroon	+	+	[31]
Pokoase 412	CC p10	Ghana	+	+	[31]
Senegal	CC p60	Senegal	+	+	[31]
Umbaneim	B	Sudan	+	+	[31]
Mara	CC p1	South Africa	+	+	[31]
Welgevonden	CC p12		+	+	[29]
Lutale	CC p6	Zambia	+	+	[31]
Sankat 430	CC p16	Ghana	+	+	[32]
Umpala	CC p6	Mozambique	+	+	[31]
MAT2-17MH1	T		+	w+	[33]
MAT2-26MH1			+	+	[33]
MAT2-32MH2			+	w+	[33]
CHOB4MH2			+	w +	[33]
CHOB25MH1			+	w+	[33]
CHA2-32MH1			+	w+	[33]
KNP51MH1	T	South Africa	+	w+	[33]
KNPC15MH1			w+	w+	[33]
KNPC2MH1			+	+	[33]
KNPC2MH2			+	+	[33]
Other tick-borne pathogens					
*A. marginale*		Argentina	–	–	
*A. phagocytophilum*	CC	USA	–	–	
*A. platys (E. platys)*	CC		–	–	
*B. bovis*		Argentina	–	–	
*B. bigemina*			–	–	
*E. canis*	CC	USA	–	–	[34]
*E. muris*	CC		–	–	[35]
PME 160055	T		+	–	
PME 160178			–	–	
PME 160277			+	–	
PME 160359			–	–	
PME 160366			+	–	
PME 160491			+	–	
*R. felis*	CC		–	–	[36]
*R. parkeri*	CC		–	–	[37]
*9 uninfected A. variegatum*	T	Guadeloupe	–	–	

*E. ruminantium* was detected from *A. variegatum* and *A. hebraeum* ticks while PME was detected from *A. americanum* ticks

*Abbreviations: T* ticks, *B* blood, *CC p* cell culture passage, *+* positive, *−* negative, *w +* weak positive

The analytical specificity of Sol1^TqM^ qPCR was evaluated using 10 tick-borne pathogens, species of *Anaplasma*, *Babesia*, *Ehrlichia* and *Rickettsia* (Table 1) [34–37]. In addition, nine DNA samples extracted from non-infected *A. variegatum* adult ticks were obtained from the tick rearing stock in the CIRAD laboratory and used as negative controls. Using BLAST, sequences of Sol1 primers and probe were checked against available *E. chaffeensis* sequences to evaluate the amplification capacity of Sol1^TqM^ qPCR in silico.

### Development of tick 16S rDNA qPCR for DNA extraction and PCR control

#### Design of 16S rDNA primers

To develop a SYBR Green qPCR, targeting the mitochondrial 16S ribosomal DNA (rDNA) gene, hereafter referred to as 16S^SG^ rDNA qPCR, the forward primer 16SF was selected from a previous paper [38] and a new reverse primer 16SR2 was designed based on eight available sequences using Primer3 [39] to obtain an optimal product size of 181 bp for the qPCR (Additional file 1: Table S1). The *Rhipicephalus*, *Amblyomma* and *Ixodes* nucleic sequences used to design the primers are described in Additional file 1.

#### Efficiency, limit of detection and reproducibility

To evaluate the analytical performance of the new 16S^SG^ rDNA qPCR, we tested 10-fold serial dilutions of DNA extracted from a single field tick (*A. variegatum*), with three replicates to define the optimal temperature and then with five replicates to obtain accurate measurements of efficiency at this temperature.

We set up a new protocol for sample preparation before DNA extraction, based on tick grinding in the TissueLyser II (Qiagen, Courtaboeuf, France). The detailed protocol for grinding and manual DNA extraction is given in Additional file 2. The amplification efficiency (E) and percentage of efficiency were calculated as described in Bustin et al. [30]. Average Ct and standard deviation (± SD) were calculated for the replicates to assess the reproducibility of the 16S^SG^ rDNA qPCR.

#### Quality control criteria

In order to set the threshold of the new 16S^SG^ rDNA qPCR, a panel of 37 field ticks (*A. hebraeum* and *A. variegatum*), collected in Mozambique and South Africa, were individually extracted on the automated platform as described below and tested. We calculated the mean Ct value for these 37 tests and set the upper limit to validate both the automated extraction of nucleic acids and the absence of inhibitors in the real-time qPCR, using the following formula:

Ct_sample_ < mean Ct value _37 ticks_ + 2 SD.

### Validation of the whole method: Performance of the automated tick DNA extraction and *pCS20* Sol1^TqM^ qPCR

#### Limit of detection

To assess the limit of detection, pools of unfed naïve tick lysates were spiked with serial dilutions of *E. ruminantium* passage 43 from infected cell cultures [28] at a concentration ranging from 6 × 10^3^ to 6 copies/reaction (details on the preparation of the samples are given in Additional file 2). The limit of detection of the new method [automated extraction on Biomek 4000 (Beckman Coulter, Villepinte, France) with the kit viral RNA and DNA (Macherey-Nagel, Hoerdt, France) in a 96-well plate format and Sol1^TqM^ qPCR) was compared in two independent assays on the same samples with the standard method based on manual DNA extraction (QiaAmp DNA minikit, Qiagen, Courtaboeuf, France) and nested PCR.

#### Performance of the manual and automated DNA extraction

The performance of the automated and manual extractions was compared using *pCS20* nested PCR and Sol1^TqM^ qPCR on 17 tick lysates spiked with *E. ruminantium* cell cultures (as described above) and lysates of 30 adult ticks moulted from nymphs experimentally engorged on infected goats. The production of these adult ticks hereafter named “experimentally infected ticks” is detailed in Additional file 3. The 47 samples were subjected to automated and manual DNA extractions in parallel and then to either Sol1^TqM^ qPCR or nested PCR (94 final results).

The quantitative and qualitative results of the nested PCR and of the Sol1^TqM^ qPCR after manual or automated extraction were converted into positive or negative, excluding any doubtful results by either nested PCR (multiple band PCR product) or by qPCR (Ct > limit of positivity). The relative specificity sensitivity and accuracy of automated extraction as compared to manual extraction were calculated as described in Additional file 4. The degree of agreement between the two extraction methods was calculated using kappa statistics [40]. Kappa values were interpreted as follows: very good agreement: ≥ 0.81; good agreement: 0.61–0.80; moderate agreement: 0.41–0.6; fair agreement: 0.21–0.4; and poor agreement: ≤ 0.20 [40].

The distributions of the Ct values generated by *pCS20* Sol1^TqM^ qPCR (*n* = 17) on experimentally infected ticks or serial dilutions of *E. ruminantium* in tick lysates both extracted automatically and manually, were represented onto a 2-D dot plot. One dot corresponds to the Cts obtained for one sample with the *pCS20* Sol1 qPCR^TqM^ on nucleic acids extracted manually (y-axis) and automatically (x-axis) [41].

#### Relative sensitivity and specificity of pCS20 Sol1^TqM^ qPCR

We determined the relative sensitivity, specificity and accuracy of the *pCS20* Sol1^TqM^ qPCR on 60 field *A. hebraeum* and *A. variegatum* adult ticks from Mozambique and South Africa [42], extracted either manually or automatically. The true status (positive or negative) of these ticks was established by the combined results of two tests, hereafter referred to as the reference method. The first test included in the reference method was the OIE gold standard *pCS20* nested PCR [11]. The second test was based on multilocus sequence typing (MLST), performed according to Cangi et al. [33].

A sample was considered as negative when both tests were scored as negative. When multiple bands were detected with the nested *pCS20* PCR but MLST was negative, the sample was also considered negative. In all other cases, the sample was scored as positive.

Relative sensitivity (Se) and specificity (Sp) were defined as the percentages of samples scored by the *pCS20* Sol1^TqM^ qPCR as positive and negative, respectively, out of all the samples scored by the reference method (*pCS20* nested PCR and MLST) as positive and negative (Additional file 4) [43]. Relative accuracy (Ac) was defined as the degree of agreement between the results obtained by the *pCS20* Sol1^TqM^ qPCR and by the reference method. The formulas used to calculate Se, Sp and Ac are detailed in Additional file 4.

Results of the Sol1^TqM^ qPCR and the *pCS20* nested PCR in combination with MLST were cross-tabulated (2 × 2 table). In addition, the kappa agreement between the two methods was determined as described above.

#### Relative sensitivity and specificity of the whole method

Finally, we compared the whole method (automated extraction + *pCS20* Sol1^TqM^ qPCR) and the standard method (manual extraction + nested *pCS20* PCR) using 47 samples: 17 samples spiked with serial 10-fold *E. ruminantium* dilutions from infected cell cultures and 30 experimentally infected ticks as described above. No doubtful samples with multiple band PCR products or Ct greater than the limit of positivity were included in the panel. Sensitivity, specificity, accuracy and kappa agreement between the two methods were determined based on positive and negative results obtained by both methods (Additional file 4).

#### Reproducibility and processing time

To estimate the reproducibility of automated DNA extraction coupled with the *pCS20* Sol1^TqM^ qPCR, *E. ruminantium* strain Gardel passage 43 was appropriately diluted and added to a supernatant of tick lysates to reach final concentrations ranging between 6 and 60 copies/reaction. Spiked tick supernatants were extracted in triplicate in separate procedures and further tested by *pCS20* Sol1^TqM^ qPCR. Last, sample processing time was estimated for automated and manual DNA extraction as well as for nested PCR and Sol1^TqM^ qPCR.

## Results

### Development of *pCS20* Sol1^TqM^ and Sol1^SG^ qPCRs

#### Optimization and efficiency of pCS20 Sol1 ^TqM^ and Sol1^SG^ qPCR

The *pCS20* Sol1^TqM^ and Sol1^SG^ qPCR efficiencies (%) were measured at different temperatures (from 50 °C to 56 °C) using 10-fold serial dilutions of *E. ruminantium* Gardel DNA in three separate experiments. With *pCS20* Sol1^SG^ qPCR, the optimal annealing temperature was 51 °C with 98.1 ± 1.9% efficiency and the mean expected temperature of dissociation was 74.2 ± 0.5 °C. The maximum PCR efficiency of Sol1^TqM^ was obtained at 55 °C with 94.4 ± 3.6%. At 54 °C and 56 °C, efficiencies were 89.1 ± 6.1% and 93.8 ± 5.8% with more variation between the tests. Consequently, the optimal PCR conditions were defined for the new *pCS20* Sol1^TqM^ and Sol1^SG^ qPCR as annealing temperatures of 55 °C and 51 °C, respectively.

With Cow^TqM^ qPCR, the use of the optimal temperature of 48 °C recommended by Steyn et al. [24], only allowed the detection of 3 × 10^4^ copies/reaction in one out of three independent assays. We then tested Cow^TqM^ qPCR at 56 °C, a temperature closer to the melting temperature of the probe, which resulted in a low PCR efficiency of 69.2 ± 3.1%.

#### Limit of detection and reproducibility of pCS20 Sol1 ^TqM^ and Sol1^SG^ qPCR

At the optimal annealing temperatures, *pCS20* Sol1^TqM^ and Sol1^SG^ qPCRs were performed on *E. ruminantium* Gardel DNA at 3 × 10^6^ to 3 copies/reaction in parallel with *pCS20* nested PCR and Cow^TqM^ qPCR. The mean Ct values of three independent runs and standard deviations are listed in Table 2. The results of the Cow^TqM^ qPCR are not shown because the detection limit achieved with this test in optimal conditions was very poor (3 × 10^3^ copies of bacteria per reaction). The limit of detection of the *pCS20* Sol1 qPCRs was better than that of *pCS20* nested PCR, with detection limits of 3 and 15 copies/reaction, respectively (Table 2). The detection threshold for *pCS20* Sol1^TqM^ was then set at a Ct of 37. With Sol1^SG^ qPCR, the Ct was 30.5 ± 1.3 and 34 ± 0.7 for 30 and 3 copies, respectively. However, a biphasic dissociation curve was found for 3 copies, highlighting the presence of both primer dimers and *pCS20* amplicons. Sol1^SG^ qPCR detected a signal for non-template control (NTC) with a Ct of 35 ± 1.1 due to primer dimers, as evidenced by a lower dissociation temperature than that of the target (data not shown). The positive threshold for Sol1^SG^ qPCR was established at 35 Ct.

**Table 2:** Limit of detection of *pCS20* Sol1^TqM^ qPCR, Sol1^SG^ qPCR and *pCS20* nested PCR

DNA *E. ruminantium* strain Gardel (copies/reaction)^a^	Sol1^TqM^ qPCR (Th = 55 °C) Ct ± SD^b^	Sol1^SG^ qPCR (Th = 51 °C) Ct ± SD^b^	*pCS20* nested PCR signal^c^
3.10^6^	17.0 ± 0.3	13.6 ± 0.6	+
3.10^5^	20.3 ± 0.1	16.8 ± 0.4	+
3.10^4^	23.6 ± 0.0	20.0 ± 0.5	+
3.10^3^	27.1 ± 0.6	23.5 ± 0.4	+
3.10^2^	31.0 ± 0.5	26.9 ± 1.0	+
30	34.2 ± 0.3	30.5 ± 1.3	w+
3	36.7^d^	34.0 ± 0.7	–
NTC	Undet	35.0 ± 1.1	–

^a^Bacterial load used for qPCR: the nested PCR samples were amplified from 1 μl of DNA, containing half the quantity from 1.5 ×10^6^ to 1.5 copies/reaction

^b^The average Ct value is indicated for each dilution (bacteria copy number) and standard deviation was derived from 3 replicates

^c^Conventional PCR done in duplicate

^d^Tested once

*Abbreviations: Th* temperature of hybridization, *w +* weak positive, *NTC* non-template control, *Undet* undetermined, *SD* standard deviation

With both *pCS20* qPCRs, the Ct standard deviations were extremely low, ranging from 0 to 1.3, demonstrating the good reproducibility of both assays (Table 2).

#### Sensitivity and specificity of pCS20 Sol1^TqM^

Twenty-six *E. ruminantium* strains from different geographic origins (Table 1) were successfully amplified by both *pCS20* Sol1^TqM^ qPCR and the gold standard test *pCS20* nested PCR. Concerning the specificity of the assay, *A. marginale*, *A. phagocytophilum*, *A. platys*, *B. bovis*, *B. bigemina*, *E. canis* and *E. muris, R. felis* or *R. parkeri* were not detected by either *pCS20* Sol1^TqM^ qPCR or *pCS20* nested PCR. Moreover, PME was not detected by *pCS20* Sol1^TqM^ qPCR whereas 4 out of 6 samples were scored positive by *pCS20* nested PCR (Table 1). Nine uninfected *A. variegatum* DNA samples from the CIRAD rearing facilities were scored negative by *pCS20* Sol1^TqM^ qPCR and *pCS20* nested PCR. Concerning *E. chaffeensis*, using BLAST sequence analysis, the Sol1 TaqMan probe did not align with the *E. chaffeensis pCS20* gene region and only 13 out of 20 nucleotides of the Sol1R primer, in the middle of the primer, matched this gene. Based on these in silico analyses, no positive results can be expected using Sol1 qPCR^TqM^ on *E. chaffeensis*.

### Development of tick 16S^SG^ rDNA qPCR as internal control for DNA extraction and PCR

#### 16S^SG^ rDNA qPCR efficiency and limit of detection

Serial dilutions (from 10^−1^ to 10^−5^) of *A. variegatum* DNA were amplified in triplicate with 16S^SG^ rDNA qPCR at different temperatures ranging from 58 °C to 61 °C. Efficiency levels ranged from 80 to 84% and did not differ significantly with the hybridization temperature. However, Ct values were higher at 60 °C and 61 °C than at 58 °C and 59 °C, with an increment of two to seven Cts depending on dilution. Furthermore, the dilution 10^−5^ of the 16S rDNA was not detected at 61 °C. The optimal hybridization temperature for the 16S^SG^ rDNA qPCR was defined as 59 °C with 84 ± 5.1% efficiency, based on five replicates. Using the serial dilutions of the positive control, the mean dissociation temperature for 16S^SG^ rDNA qPCR was 72.1 ± 0.2 °C (*n* = 5).

#### Quality control of automated DNA extraction and reproducibility

A panel of 37 field samples were subjected to automated DNA extraction, and all the samples were successfully amplified by 16S^SG^ rDNA qPCR, with a mean Ct of 23.3 ± 2.8, suggesting that the DNA extracted by the robot was of good quality and no PCR inhibitors were present (data not shown). The acceptable limit of Ct attesting to the good DNA quality was set at 29, corresponding to mean Ct value of the 37 field samples +2SD. The reproducibility of the whole method (DNA extraction and 16S^SG^ qPCR) was evaluated, and less than 4 Ct variation was found in the same tick lysate extracted in four different repetitions.

### Validation of the whole method (automated tick DNA extraction and *pCS20* Sol1^TqM^ qPCR)

#### Limit of detection

The automated DNA extraction followed by *pCS20* Sol1^TqM^ qPCR enabled the detection of *E. ruminantium* from infected cell cultures down to six copies/reaction with Ct = 37.6 ± 1. These results were obtained in two independent assays (data not shown). When the same samples underwent manual extraction and *pCS20* nested PCR, the detection limit of *E. ruminantium* was the same, with six copies/reaction.

#### Comparison of the performances of automated and manual DNA extraction

Automated and manual DNA extractions were compared in a total of 47 samples screened either by *pCS20* Sol1^TqM^ qPCR or nested *pCS20* as described in Methods. It is worth noting that only 30% (9/30) of experimentally infected ticks were found to be positive by nested PCR. The relative sensitivity, specificity and accuracy of automated extraction compared to manual extraction was 84.1%, 88% and 86.2%, respectively (Table 3). Kappa agreement between both extraction methods was 72%.

**Table 3:** Relative sensitivity and specificity of automated DNA extraction compared with manual DNA extraction based on screening of samples tested either by nested *pCS20* PCR or Sol1^TqM^ qPCR. Results (positive and negative *E. ruminantium* samples, Se, Sp and Acc of the automated extraction method) obtained on tick lysates spiked with *E. ruminantium* serial dilutions (*n* = 17) and experimentally infected ticks (*n* = 30) extracted in parallel by manual and automatic extraction and tested either by nested PCR or *pCS20* Sol1^TqM^ qPCR are shown. The kappa test was 72%

		Manual extraction		Total	Se (%)	Sp (%)	Ac (%)
		+	–				
Automated extraction	+	37	6	43	84.1	88.0	86.2
	–	7	44	51			
Total		44	50	94			

*Abbreviations: Se* relative sensitivity, *Sp* relative specificity, *Ac* accuracy

**Fig. 1:**
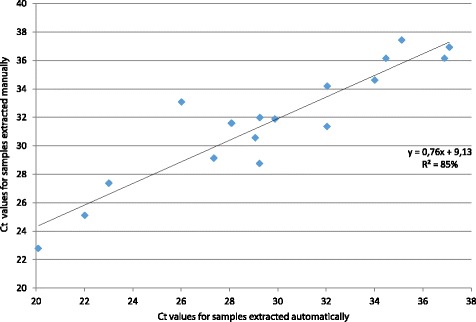
Comparison of Ct values obtained by Sol1^TqM^ qPCR for tick lysates spiked with *E. ruminantium* serial dilutions and experimentally infected ticks extracted through automated and manual techniques (*n* = 17)

Six and seven samples were not detected as positive for *E. ruminantium* with manual and automated extractions, respectively (Table 3). Since the possibility of contamination between samples was excluded by repeated tests, these 13 samples were “true positives”, showing that the two methods of extraction have similar sensitivity. A comparison of Ct values obtained with tick lysates spiked with *E. ruminantium* serial dilutions and experimentally infected ticks extracted automatically and manually in parallel is shown in Fig. 1. A good correlation was observed (*R* ^2^ = 85%) and the Ct values were slightly better under automated DNA extraction (-1.96 Ct, *P* < 0.001). In conclusion, the performance of the automated DNA extraction method is the same as that of manual extraction for the detection of *E. ruminantium* by *pCS20* Sol1^TqM^ qPCR and nested qPCR.

#### Relative sensitivity and specificity of the pCS20 Sol1^TqM^ qPCR

Compared to the reference method (*pCS20* nested PCR and combined with MLST), the relative sensitivity and specificity of the *pCS20* Sol1^TqM^ qPCR was 75.8 and 85.2%, respectively with 80% accuracy (Tables 4, 60 field ticks extracted using manual and automated methods). Out of the 8 false negative and 4 false positive samples, 4 were scored positive and 2 negative, respectively, by the two tests comprising the reference method. Of the 4 false positive samples, the other 2 samples displayed multiple bands by nested PCR and were scored negative by MLST. The Cts obtained for these 4 false positives ranged between 34.2 and 36.7. Out of the 25 true positive and 23 true negative samples, only 19 (76%) were scored positive and 5 (22%) were negative, respectively, by the two tests comprising the reference method. Concerning the true positive samples, out of the 6 remaining samples, 3 were scored positive by nested PCR and negative by MSLT and 3 were scored positive by MLST (corresponding to samples with partial amplification of at least one MLST gene) and negative by nested PCR. The mean Cts for these 6 samples was 37.1 ± 0.6, at the limit of detection. Out of the 23 true negative samples, 18 (78%) displayed multiple bands by nested PCR and were scored negative by MLST.

**Table 4:** Relative sensitivity and specificity of *pCS20* Sol1^TqM^ qPCR compared with the *pCS20* nested PCR and MLST, which were combined as the reference method. Results obtained from 60 field-collected ticks are shown. The kappa test was 60%

		Nested PCR + MLST		Total	Se (%)	Sp (%)	Ac (%)
		+	–				
Sol1^TqM^ qPCR	+	25	4	29	75.8	85.2	80.0
	–	8	23	31			
Total		33	27	60			

*Se* relative sensitivity, *Sp* relative specificity, *Ac* accuracy

The kappa statistics for the *pCS20* Sol1™ qPCR/ *pCS20* nested PCR + MLST comparisons were 60%, demonstrating fair to good agreement between the tests.

#### Relative sensitivity and specificity of the whole method

The relative sensitivity and specificity of the whole method (automated DNA extraction + *pCS20* Sol1^TqM^ qPCR) compared with manual extraction + *pCS20* nested, were 76.2% and 73.1%, respectively (Table 5), with 7 and 5 additional positive samples detected by the new method and the standard method, respectively. Four out of the 7 false positive samples had a Ct of 37, at the threshold. The 5 false negative samples were clearly positive with the nested *pCS20* PCR. The kappa value for this analysis was 49%, demonstrating moderate agreement between the tests.

**Table 5:** Relative sensitivity and specificity of automated extraction + *pCS20* Sol1^TqM^ qPCR compared with manual extraction + *pCS20* nested PCR. Results obtained on 17 *E. ruminantium* serial dilutions and 30 experimentally infected ticks. The kappa test for this analysis was 49%

		Manual extraction + *pCS20* nested PCR		Total	Se (%)	Sp (%)	Ac (%)
		+	–				
Automated extraction *+*Sol1^TqM^ qPCR	+	16	7	23	76.2	73.1	74.5
	–	5	19	24			
Total		21	26	47			

*Se* relative sensitivity, *Sp* relative specificity, *Ac* accuracy

#### Reproducibility of the automated DNA extraction and pCS20 Sol1 ™ qPCR

The reproducibility of the whole method (automated extraction + *pCS20* Sol1^TqM^ qPCR) on independent triplicates was high, as demonstrated by the low standard deviation of Ct values: Ct = 33 ± 0.7 (CV = 2.1%) and Ct = 36.8 ± 1.3 (CV = 3.5%) for samples with 60 and 6 *E. ruminantium* copies/reaction, respectively.

#### Sample processing time

The total time required to extract DNA/RNA from 96 tick samples automatically was four hours, compared to 2.5 days for manual DNA extraction in our facilities. The first step in the preparation of tick lysates requires manual pipetting of reagents and crushed tick lysates. However, the use of a Tissue Lyser allowed simultaneous processing of 48 samples to obtain tick lysates. In addition, the nested PCR alone takes 1.5 days to perform, whereas the Sol1^TqM^ qPCR can be completed in four hours. Furthermore, the risk of contamination is substantially reduced with the Sol1^TqM^ qPCR.

## Discussion

In our conditions, the use of *pCS20* Cow^TqM^, described by Steyn et al. [24], was inefficient throughout the present study (PCR efficiency of 69.2%). As we successfully optimized Sol1^TqM^ qPCR in parallel, using the same DNA and reagents, the lack of efficiency cannot be explained by the presence of inhibitors or deficient reagents. Moreover, Cow^TqM^ primers and probes hybridized entirely on the Gardel *pCS20* gene region, thereby limiting the impact of genetic variability on the result. The reason for unsuccessful implementation of *pCS20* Cow^TqM^ in our facilities remains unclear.

Both qPCR *pCS20* Sol1 using SYBR Green and TaqMan probe chemistries for detection of *E. ruminantium* were more than 94% efficient. Even though the specificity and sensitivity of Sol1^SG^ qPCR were not evaluated as thoroughly as those of Sol1^TqM^ qPCR, this new qPCR could be a cheaper alternative for the detection of *E. ruminantium* in laboratories in low income countries. Similar PCR efficiencies obtained for Sol1^TqM^ at 55 °C and 56 °C also confirmed the robustness of the assay.

The limit of detection of the *pCS20* Sol1^TqM^ and Sol1^SG^ is three *E. ruminantium* copies per reaction, which is better than the *pCS20* nested PCR (15 copies per reaction in this study, 6 copies in another study [18]), the Cow^TqM^ qPCR (14 copies per reaction in the work of Steyn et al. [24], 3000 copies per reaction in our hands). It is also better than the new dual-plex qPCR targeting PME/*E. ruminantium* (10 copies per reaction) [27] and the new multiple pathogen detection tool enabling the detection of eight *Ehrlichia* species, including *E. ruminantium* (5 copies per reaction) [25]. In the present study, the positivity thresholds were set at 37 and 35 cycles for Sol1^TqM^ and Sol1^SG^ qPCRs, respectively. The positivity threshold for Sol1^TqM^ qPCR was confirmed by the results obtained with the whole method (automated extraction and Sol1^TqM^ qPCR) in which three samples with six copies had a mean Ct value(s) of 36.8 ± 1.3. Detection of such a low number of copies is important as it enables the detection of the low bacterial loads as frequently observed in infected ticks. Moreover, *pCS20* Sol1^TqM^ qPCR was able to detect *E. ruminantium* in blood samples of three experimentally infected goats during hyperthermia (data not shown). Thus, *pCS20* Sol1^TqM^ can also be recommended for the diagnosis of heartwater from blood samples.

In contrast to Cow^TqM^ qPCR, which cross-reacted with *E. chaffeensis* and *E. canis* [23]*,* the new *pCS20* Sol1^TqM^ qPCR did not cross-react with other tick-borne pathogens including PME. It was also shown to detect 26 different *E. ruminantium* strains from a wide range of geographic origins including the Caribbean, West, East, and southern Africa. In a previous study, 797 *Amblyomma* ticks collected in Mozambique and southern Africa were extracted using the whole method (automated DNA extraction and Sol1^TqM^ qPCR), described in this paper [33]. Positive samples obtained with Sol1^TqM^ qPCR were typed by MLST, enabling the identification of genetic groups G1 and G2 including G2A, G2B, G2C, G2D subgroups that covered the wide genetic diversity of *E. ruminantium*.

The performance of *pCS20* Sol1^TqM^ qPCR was tested by comparing the results of detection using African ticks collected in the field and infected tick lysates with those obtained with *pCS20* nested PCR and MLST. Among the 25 true positive samples, six samples were scored positive only by nested PCR or by MLST and had high Cts by *pCS20* Sol1^TqM^ qPCR. These results confirmed the ability of *pCS20* Sol1^TqM^ qPCR to detect low bacterial loads and some strains that could not be amplified by nested PCR or MLST. Moreover, among the true negative samples, 78% of doubtful status (PCR products with multiple bands) identified by nested *pCS20* PCR and scored negative by MLST, were also scored negative by Sol1^TqM^ qPCR, showing that Sol1^TqM^ qPCR gives reliable negative results whereas the nested *pCS20* gives inconclusive results.

As we observed no cross-reactions with other tick-borne pathogens, including closely related species, and a higher limit of detection, it is possible that the positive results obtained with *pCS20* Sol1^TqM^, which were negative with *pCS20* nested PCR and MLST, were truly infected samples.

The specificity and sensitivity for *E. ruminantium* detection of new PCR method therefore appears to be better than that of nested *pCS20* PCR and Cow^TqM^ qPCR. Moreover, we demonstrate that this PCR method enabled the detection of a large number of *E. ruminantium* strains in contrast to Cow^TqM^ qPCR which was only able to successfully detect 15 *E. ruminantium* strains.

Another advantage of our assay is it does not detect PME allowing its use in the USA with a low risk of false positives, avoiding the use of a dual-plex TaqMan qPCR to differentiate PME from *E. ruminantium* [27]. The recently published new multiple Ehrlichia detection tool [25] is promising since it detects up to eight Ehrlichia species including *E. ruminantium*. However, it was only tested on five *E. ruminantium* strains, and not on *E. ruminantium* positive field samples, so further studies are needed to confirm its possible extensive use on tick samples in the field.

We successfully optimized another qPCR targeting the 16S rDNA tick gene. It can be used for several tick genera or species including *Amblyomma*, *Rhipicephalus* and *Ixodes* [44, 45]. The 16S^SG^ rDNA qPCR is a powerful method for DNA extraction and quality control, DNA quantification and assessment of the presence of PCR inhibitors that complements standard methods using gel migration and nanodrop.

Furthermore, using the 16S qPCR for DNA quality evaluation circumvents the limitations of photometric and fluorometric methods for DNA quality assessment when the viral RNA and DNA Macherey-Nagel kit is used. These limitations are due to the presence of an RNA carrier, which leads to overestimation of the amount of nucleic acids.

We observed a similar performance between the automated and manual extractions of DNA from ticks whatever the PCR used downstream, excepting for samples with low loads of *E. ruminantium.* For these latter samples, the automated DNA extraction and Sol1^TqM^ qPCR appeared to perform better than conventional methods. We also showed that the whole method (automated DNA extraction and Sol1 qPCR^TqM^) was highly reproducible. In our laboratory, the processing and testing of 96 samples with the automated method requires only one day of work by one technician whereas with the OIE standard method, it would take three and a half days. Although the first step in the preparation of the tick samples (washing and grinding) before extraction was not automated, it was significantly shortened by the use of a TissueLyser II. We thus conclude that, depending on the number of samples and their bacterial load, DNA extraction methods may be interchangeable.

The commercial kit produced by Macherey-Nagel, which was adapted for automated extraction of tick samples in our experiments, has the advantage of extracting both DNA and RNA from viruses as well as bacteria. This kit was also tested on the avian influenza virus in our laboratory and its virus detection performance was similar (data not shown). The automated DNA extraction method may thus also be useful for the screening of other pathogens, including viruses in ticks, and possibly the genetic characterization of ticks and co-evolution studies.

## Conclusions

The whole method, i.e. automated DNA extraction, adapted for tick samples and coupled with the new Sol1 qPCR, is more sensitive, specific and reproducible and reduces the risk of contamination. Using this method, *E. ruminantium* in ticks is also detected faster than any other existing methods including the OIE reference method based on manual DNA extraction and *pCS20* nested PCR. It will be a useful tool for screening a large number of ticks for *E. ruminantium*. Independently of DNA extraction, the *pCS20* Sol1^TqM^ and Sol1^SG^ qPCRs will also be valuable for *E. ruminantium* detection in ticks or in blood samples from clinically suspicious ruminants. Given there is no cross reaction with the endemic PME or *E. chaffeensis*, this could be of particular interest in the parts of the American continent that are currently free, such as mainland USA. Likewise, this high-throughput DNA extraction method using a virus RNA/DNA extraction kit validated for tick samples has potential for genetic studies on ticks, and for field screening of other bacteria and viruses in ticks.

## Additional files

Additional file 1: Text. PCR protocol for the detection of *E. ruminantium.* **Table S1.** Primers and probes for *pCS20* Sol1^TqM^, Sol1^SG^, Cow™ and 16S^SG^ qPCRs. (DOC 48 kb)

Additional file 2: Text. Protocol of extraction of tick DNA. (DOCX 12 kb)

Additional file 3: Text. Adult ticks from nymphs engorged on experimentally infected goats. (DOCX 11 kb)

Additional file 4: Text. Formula for relative sensitivity, specificity and accuracy. (DOCX 12 kb)

## Abbreviations

Ac: Accuracy
B: Blood
CC p: Cell culture passage
Ct: Cycle threshold
FN: False negative
FP: False positive
MLST: Multi-locus sequence typing
NTC: Non-template control
PME: Panola Mountain *Ehrlichia*
qPCR: Quantitative polymerase chain reaction
RLB: Reverse line blot
Se: Sensitivity
^SG^: SYBR green
Sp: Specificity
T: Ticks
Th: Temperature of hybridization
TN: True negative
TP: True positive
^TqM^: TaqMan
Undet: Undetermined
w +: Weak positive.
