# Supplementary material for: Efficient high-throughput molecular method to detect Ehrlichia ruminantium in ticks
Source: Parasit Vectors. 2017 Nov 13;10:566. doi: 10.1186/s13071-017-2490-0 (PMC5683323; doi:10.1186/s13071-017-2490-0)
Supplement: Supplementary file 1 — Text. PCR protocol for the detection of E. ruminantium. Table S1. Primers and probes for pCS20 Sol1TqM, Sol1SG, Cow™ and 16SSG qPCRs. (DOC 48 kb) [file 13071_2017_2490_MOESM1_ESM.doc]

**Additional file 1: PCR protocol for the detection of *E. ruminantium***

Real-time PCR was performed on 7000 and 7500 System thermocyclers (Applied Biosystems, Villebon sur Yvette, France) and results were analysed using 7500 System SDS Software (Applied Biosystems, Villebon sur Yvette, France). In all the runs, positive and negative standard controls consisting of *E. ruminantium* Gardel strain and water were included.

**1. Design of *pCS20* Sol1** **primers and probes**

For the design of Sol1 primers and probes, the most conserved region of *E. ruminantium*, *pCS20*, was identiﬁed through multiple alignments of nucleotide sequences from 13 strains available in GenBank:

Kwanyanga (AY236063), Mara87/7 (AY236064), Sankat430 (AY236065), Senegal (AY236066), Pokoase (AY236067), Mali (AY236068), Kumm1 (AY236069), Welgevonden (AY236058), Ball3 (AY236059), Vosloo (AY236060), Gardel (AY236061), Crystal Spring (AY455275) and Blaauwkrantz (AY236062).

The Sol1 TaqMan probe for *pCS20* Sol1 qPCR was designed to target this conserved region using the software LightCycler® Probe Design (Table S1).

Hybridization for Sol 1 primers was 100% and there were only 2 mismatches for the probe.

**2. Real-time PCR *pCS20* Sol1^TqM^, Sol1^SG^ and Cow^TqM^**

The *pCS20* Sol1^SG^ qPCR assay with SYBR Green was performed using the Power SYBR® Green PCR Master Mix (Life Technologies, Courtaboeuf, France). Each 25 µl reaction mixture contained 250 nM each of forward and reverse primer and standard concentrations of SYBR Green Dye, ROX™, AmpliTaq Gold® DNA Polymerase LD, dNTPs with dUTP/dTTP blend, optimised buffer components, 12.5 µl of distilled water and 2.0 µl sample DNA. The thermocycling conditions were 2 minutes at 50°C for activation of Uracil-N-Glycosylase, 10 minutes at 95°C for inactivation of Uracil-N-Glycosylase (UDG) and activation of the AmpliTaq Gold® DNA polymerase and 40 cycles of 15 seconds at 95°C for denaturation and 60 seconds at 51^o^C for annealing and extension. Following amplification, the specificity of the PCR products was confirmed by analysis of dissociation curves.

The *pCS20* Sol1^TqM^ qPCR assay with a TaqMan probe was performed using the TaqMan® Universal PCR Master Mix (Life Technologies, Courtaboeuf, France). The final reaction contained 250 nM each of forward and reverse primer, 200 nM of the probe, ROX™, AmpliTaq Gold® DNA Polymerase LD, dNTPs with dUTP/dTTP blend, optimised buffer components, 12.5 µl of distilled water and 2.0 µl sample DNA. The qPCR was run in a final volume of 25 µl of which 2 µl corresponded to the sample DNA. The thermocycling conditions were 2 minutes at 50°C, 10 minutes at 95°C denaturation and 40 cycles of 15 seconds at 95°C for denaturation and 60 seconds at 55^o^C for annealing and extension.

Both qPCR master mix contained internal passive reference dye ROX™ and Uracil-N-Glycosylase (UDG).

The Cow^TqM^ qPCR assay developed by Steyn et al [24] with a TaqMan probe was run using the TaqMan® Universal PCR Master Mix (Life Technologies, Courtaboeuf, France). The final reaction contained the reagents, volumes and thermocycling conditions described above, but it was tested at annealing temperatures of 48^o^C and 56^o^C.

**3. Real-time PCR amplification for DNA quality control of ticks based on the 16S rDNA**

**3.1. Design of 16S rDNA primers**

The forward primer 16SF was selected from a previous paper [32], and a new reverse primer 16SR2 was designed to obtain an optimal product size of 181 bp for qPCR (Table 1). The design of 16SR2, using Primer3 [33], was based on the alignment of the partially conserved region of 16S rDNA from 8 tick species and strains available in GenBank: *Rhipicephalus geigyi* strain C7M (KF569942.1), *Ixodes minor* (KF793047.1), *Amblyomma boeroi* voucher INTA 2185 (JN828797.1), *A. glauerti* (AGU95853), *A. maculatum* clone TD02-239.T16s (AY375442.1, *I. minor* (KF793047.1), *A. glauerti* (AGU95853) and *A. maculatum* clone TD02-239.T16s (AY375442.1).

**3.2. Cycling conditions for 16S rDNA**

The 16S^SG^ rDNA qPCR was optimised by testing a temperature gradient from 58^o^C to 61^o^C and different concentrations of primer. The qPCR assays were performed using Power SYBR® Green PCR Master Mix (Life Technologies, Courtaboeuf, France). Each 25 µl reaction mixture contained 250 nM each of forward and reverse primer and standard concentrations of SYBR Green Dye, ROX™, AmpliTaq Gold DNA Polymerase LD, dNTPs with dUTP/dTTP blend, optimised buffer components, 12.5 µl of distilled water and 2.0 µl sample DNA. Either Platform 7000 or 7500 (Applied Biosystems) was used for DNA ampliﬁcation. The thermocycling conditions were set at 2 min at 50°C for activation of UDG, 10 minutes at 95°C for inactivation of UDG and activation of the AmpliTaq Gold® DNA polymerase, and 40 cycles of 15 seconds at 95°C and 60 seconds at 59°C. The qPCR controls included a positive control (*A. variegatum* DNA) and a negative control (water). The results of the 16S^SG^ rDNA qPCR were analysed by 7500 System SDS Software (Applied Biosystems, Villebon sur Yvette, France).

**Table S1** Primers and probes for *pCS20* Sol1^TqM^, Sol1^SG^, Cow^TM^ and 16S^SG^ qPCRs

| **qPCR name** | | **Primer/ Probe** | **Sequence (5' → 3')** | **Melting T (°C)** | **Optimal Th (°C)** | **Product size (bp)** | **Reference** |
| --- | --- | --- | --- | --- | --- | --- | --- |
| Sol1^SG/TqM^ | Sol1 F  Sol1 R | | ACA AAT CTG GYC  CAG ATC AC  CAG CTT TCT GTT CAG  CTA GT | 56.4  56.5 | 51 | 110 pb | This study |
| Sol1^TqM^ | Sol1^TM^ probe | | **6-FAM**-ATC AAT TCA CAT GAA ACA TTA CATG CAA CTG G- **BHQ1** | 72.3 | 55 |  |  |
| Cow^TqM^ | Cow F  Cow R | | CAA AAC TAG TAG AAA TTG CACA  TGC ATC TTG TGG TGG TAC | 56.3  57.9 | 48 | 226 bp | Adapted  [24] |
|  | Cow^TM^ probe | | **FAM** -TCC TCC ATC AAG ATATATAGC ACC TAT TA-**TAM** | 63.5 | 58 |  |  |
| 16S^SG^ | 16SF | | CTGCTCAATGATTTTTTAAATTGCTGTGG | 61 | 59 | 181pb | Adapted  [32] |
|  | 16SR2 | | TCTTAGGGTCTTCTTGTCDTTAATTTT | 66 | 59 |  | This study |
