# Supplementary material for: Efficient high-throughput molecular method to detect Ehrlichia ruminantium in ticks
Source: Parasit Vectors. 2017 Nov 13;10:566. doi: 10.1186/s13071-017-2490-0 (PMC5683323; doi:10.1186/s13071-017-2490-0)
Supplement: Supplementary file 2 — Text. Protocol of extraction of tick DNA. (DOCX 12 kb) [file 13071_2017_2490_MOESM2_ESM.docx]

**Additional file 2: Protocol for extraction of tick DNA**

**1. Preparation of tick samples before DNA extraction**

The ticks were ground using a TissueLyser II (Qiagen, Courtaboeuf, France) following the steps described above. A 5 mm steel bead was added to a 2 ml Eppendorf tube (Eppendorf, Montesson, France) containing a tick, and kept at -80^o^C for at least 2 hours or preferably overnight. The tick was disrupted twice, for 2 minutes, in a 2 x 24 tube format TissueLyser II at an oscillation frequency of 30 Hz. The mashed tick was then resuspended in 450 µl of sterile PBS, vortexed and centrifuged twice at 8000 rpm for 30 sec and the supernatant was recovered for nucleic acid extraction.

**2. Manual DNA extraction from ticks**

DNA from the tick lysates was extracted with the QiaAmp DNA minikit (Qiagen, Courtaboeuf, France) according to the supplier’s instructions, with a slight adjustment. Tick samples weighing 25 to 40 mg were lysed with 180 µl of buffer ATL and 20 µl of RNase A at 20 mg/ml (Sigma-Aldrich, France).

**3. Automated DNA extraction from ticks**

The automated DNA extraction was performed on 150 µl of the spiked or non-spiked tick supernatant, using the Biomek 4000 automated liquid handling robot (Beckman Coulter, Villepinte, France) and the kit viral RNA and DNA (Macherey-Nagel, Hoerdt, France) in a 96-well plate format, according to the supplier’s instructions plus adding Proteinase K treatment. Finally, nucleic acids were eluted with 100 µl nuclease-free water then with 50 µl nuclease-free water. After extraction, the plate containing the nucleic acids was stored at -20°C until use. For the purpose of comparison, 150 µl of the same samples were extracted manually, in parallel, as described in the previous section with a slight modification (150 µl of sample instead of 180 µl).
