# Supplementary material for: Efficient high-throughput molecular method to detect Ehrlichia ruminantium in ticks
Source: Parasit Vectors. 2017 Nov 13;10:566. doi: 10.1186/s13071-017-2490-0 (PMC5683323; doi:10.1186/s13071-017-2490-0)
Supplement: Supplementary file 3 — Text. Adult ticks from nymphs engorged on experimentally infected goats. (DOCX 11 kb) [file 13071_2017_2490_MOESM3_ESM.docx]

**Additional file 3: Adult ticks from nymphs engorged on experimentally infected goats**

The experimental infection of nymphs on goats was done at CIRAD herd facilities, according to internationally approved OIE standards. The experimental procedure was authorized by the director of Guadeloupe Veterinary Services on behalf of the Prefect of Guadeloupe (authorization number: A-971-18-01). *Amblyomma variegatum* nymphs were first engorged before the period of hyperthermia on goats that were experimentally challenged with the *E. ruminantium* strain, Bekuy 255. The moulted adults were preserved in 70% ethanol before further DNA extraction.
