# Supplementary material for: Efficient high-throughput molecular method to detect Ehrlichia ruminantium in ticks
Source: Parasit Vectors. 2017 Nov 13;10:566. doi: 10.1186/s13071-017-2490-0 (PMC5683323; doi:10.1186/s13071-017-2490-0)
Supplement: Supplementary file 4 — Text. Formula for relative sensitivity, specificity and accuracy. (DOCX 12 kb) [file 13071_2017_2490_MOESM4_ESM.docx]

**Additional file 4: Formula for relative sensitivity, specificity and accuracy**

Relative Sensitivity:

Se = 100*TP/(TP+FN) %, where TP stands for true positive (e.g. positive in both tests) and FN stands for false negative (e.g. negative after automated extraction but positive after manual extraction either tested by nested PCR or *pCS20* Sol1^TqM^ qPCR; negative with *pCS20* Sol1^TqM^ qPCR but positive with the reference method; negative after automated extraction and *pCS20* Sol1^TqM^ qPCR but positive after manual extraction and nested PCR).

Relative specificity:

Sp = 100*TN/(TN+FP)%, where TN stands for true negative (e.g. negative in both tests) and FP stands for false positive (e.g. positive after automated extraction but negative after manual extraction either tested by nested PCR or *pCS20* Sol1^TqM^ qPCR; positive with *pCS20* Sol1^TqM^ qPCR and negative in the reference method; positive after automated extraction and *pCS20* Sol1^TqM^ but negative after manual extraction and nested PCR).

Relative Accuracy:

Ac=100*(TP+TN)/(TP+TN+FP+FN)%
